# Support vector machine prediction of individual Autism Diagnostic Observation Schedule (ADOS) scores based on neural responses during live eye-to-eye contact

**DOI:** 10.1038/s41598-024-53942-z

**Published:** 2024-02-08

**Authors:** Xian Zhang, J. Adam Noah, Rahul Singh, James C. McPartland, Joy Hirsch

**Affiliations:** 1 Brain Function Laboratory, Department of Psychiatry, Yale School of Medicine, 300 George St., Suite 902, New Haven, CT USA; 2 Wu Tsai Institute, Yale University New Haven, New Haven, CT 06511 USA; 3 Yale Child Study Center, Nieson Irving Harris Building, 230 South Frontage Road, Floor G, Suite 100A, New Haven, CT 06519 USA; 4 Center for Brain and Mind Health, Yale School of Medicine, New Haven, CT 06511 USA; 5 Department of Neuroscience, Yale School of Medicine, New Haven, CT 06511 USA; 6 Department of Comparative Medicine, Yale School of Medicine, New Haven, CT 06511 USA; 7 Department of Medical Physics and Biomedical Engineering, University College London, London, WC1E 6BT UK

**Keywords:** Autism spectrum disorder (ASD), Eye-to-eye contact, Nested cross-validation, Functional near-infrared spectroscopy (fNIRS), Support vector machine (SVM), Biomarker, ADOS, Predictive markers, Visual system, Dynamical systems

## Abstract

Social difficulties during interactions with others are central to autism spectrum disorder (ASD). Understanding the links between these social difficulties and their underlying neural processes is a primary aim focused on improved diagnosis and treatment. In keeping with this goal, we have developed a multivariate classification method based on neural data acquired by functional near infrared spectroscopy, fNIRS, during live eye-to-eye contact with adults who were either typically developed (TD) or individuals with ASD. The ASD diagnosis was based on the gold-standard Autism Diagnostic Observation Schedule (ADOS) which also provides an index of symptom severity. Using a nested cross-validation method, a support vector machine (SVM) was trained to discriminate between ASD and TD groups based on the neural responses during eye-to-eye contact. ADOS scores were not applied in the classification training. To test the hypothesis that SVM identifies neural activity patterns related to one of the neural mechanisms underlying the behavioral symptoms of ASD, we determined the correlation coefficient between the SVM scores and the individual ADOS scores. Consistent with the hypothesis, the correlation between observed and predicted ADOS scores was 0.72 (*p* < 0.002). Findings suggest that multivariate classification methods combined with the live interaction paradigm of eye-to-eye contact provide a promising approach to link neural processes and social difficulties in individuals with ASD.

## Introduction

Autism spectrum disorder (ASD) represents a heterogeneous group of neurodevelopmental conditions marked by nontypical social communication behaviors (American Psychiatric Association, 2013). The heterogeneity of ASD has been well established by neuroimaging and genetic research^1,2^. These differences are thought to arise early in development^3,4^, and are frequently associated with stereotypical behaviors including reluctance to make eye contact^5,6^. Autism spectrum disorder affects approximately 1% of the population^7^, and is associated with a global burden of social difficulties which amplifies the importance of early detection and intervention^8^. However, both detection and intervention are challenged by the heterogeneity across individuals which has contributed to a lack of quantitative methods for diagnosis, a theoretical framework to model the underlying neural mechanisms, and evidence-based treatments.

Currently, the diagnosis of ASD is based on metrics that quantify behavioral observations rather than neural or physiological findings. Although a large body of neuroimaging investigations, primarily based on functional magnetic resonance imaging, fMRI, has focused on understanding the relationship between neural responses and social difficulties, the accumulated piecemeal findings have failed to produce a unified understanding of the underlying causes. Nonetheless, the evidence for neural-based social difficulties in ASD is well-documented by fMRI, electroencephalography (EEG), behavioral findings, and eye-tracking investigations. Many prior investigations of the neural systems in ASD have employed simulated faces such as pictures and videos to investigate social difficulties. Faces are thought to be the conduits of emotional communication and primary sources of cues that guide social interaction. Therefore, faces are a frequent choice for a social stimulus. For example, a meta-analysis of 48 investigations of simulated faces document emotional face recognition difficulty in autism^9^.

These findings are consistent with a similar review of behavioral and neuroimaging studies that also document facial recognition and emotional difficulties in autism spectrum disorders based on evidence from eye tracking, electrophysiological, and brain imaging studies that show altered face-related neural patterns, delayed event-related potential components in response to faces, and atypical activity in emotion processing circuitry^10^. Atypical processing of social information in faces has also been reported in ASD consistent with a reduced ability to link visual perception of faces and typical social behavior^11^. Further, investigations of the neural circuitry of emotional face processing and autism spectrum disorders report variations in connectivity between the amygdala and the ventromedial prefrontal cortex (a network implicated in emotional modulation) consistent with both emotion and face processing disturbances in ASD^12^.

Visual gaze is widely appreciated for its role in social interactions^13^. An eye-tracking study on facial emotion recognition tasks in adults with high functioning autism spectrum disorders reported significant differences in fixation time between typical controls and participants with ASD particularly when judging complex emotions^14^. Consistent with these findings different eye tracking patterns in ASD have been reported in toddler and preschool children^15^, and eye-movement patterns have also been shown to be altered in adults with ASD when viewing faces^16,17^. This large body of representative evidence suggests that multiple aspects of face processing are atypical in autism including gaze processing, memory for facial identity, and recognition of emotion expressed by facial configuration. In a comprehensive review of autism and the development of face processing, the roles of the superior temporal sulcus and fusiform face area were highlighted as regions associated with the neural basis of face processing anomalies in autism^18^. Nonetheless, the precise mechanisms for altered face and gaze processing in ASD remain unknown and a long-standing obstacle for a comprehensive understanding of the neural and behavioral links between social difficulties and the underlying neural substrates in ASD. The paucity of available treatment approaches, in part, reflects this knowledge gap.

Although this large body of prior findings is focused on social difficulties in ASD, the actual investigations are generally based on non-interactive social stimuli and tasks. For example, functional magnetic resonance imaging, fMRI, is a primary methodology to investigate neural properties characteristic of ASD. However, in fMRI neural information is acquired during non-interactive and stationary conditions due to the solitary and confined neuroimaging environment. A solution to this problem is enabled by current dyadic paradigms that employ functional near infrared spectroscopy, fNIRS, as the neuroimaging technology enabling an ecologically valid neurobiological approach.

Here we introduce a paradigm shift that builds on prior fMRI and fNIRS work to investigate social difficulties in real world and everyday situations as experienced by individuals with ASD. Prior investigations of *live* face gaze have revealed activity in neural systems not observed in conventional static and simulated face stimuli. These regions include the angular gyrus, superior temporal gyrus, and the supramarginal gyrus in the right hemisphere^19–21^. Further, eye-to-eye contact investigated in the live dyadic paradigm has been shown to specifically activate the dorsal visual stream including dorsal parietal regions such as the somatosensory association cortex^19^.

A similar investigation comparing eye tracking and neural systems compared responses of typical participants and those with autism spectrum disorder. Findings revealed a large system variation in neural patterns between the two groups. Specifically, whereas in the TD group, the dorsal stream, i.e. the somatosensory associated cortex, increased activity during eye-to-eye contact, in the ASD group, the ventral stream, i.e., the superior temporal gyrus and lateral occipital cortex, increased activity during eye-to-eye contact^19^. These differences, however, are group contrast-based findings that fall short of predictions for individual patient diagnoses. These observed findings and the goal to advance approaches that have predictive value based on neural and visual responses have led to the hypothesis that machine learning approaches in combination with the new dyadic paradigm with live face interaction may yield an impactful advance in characterizing the neural and behavioral components of social difficulties in individuals with autism.

The diversity of symptomatology in individuals with ASD challenges both diagnosis and evidence-based treatments. Here we suggest that a multivariate approach may address this diversity and provide an impactful advance toward unification of neural and behavioral domains. Conventional univariate analyses (such as the general liner model, GLM, based on group comparisons of magnetic resonance imaging, fMRI, data) do not provide individual level classifications due to both limited signal to noise ratio for each channel and variability with respect to patterns of neural activity. On the other hand, multivariate analysis tools, such as machine learning, combine many data features to characterize the heterogeneous neurobiology of ASD^22–24^. Here we report an application of machine learning to investigate the neural underpinnings of social symptomatology in ASD based on live interpersonal interactions during eye-to-eye contact.

Multivariate classification has been previously utilized in multiple studies on large ASD data sets including resting state^25^ and structural MRI data^26^. On one dataset, The Autism Brain Imaging Data Exchange [ABIDE], 60%, 67% and 70% accuracy has been achieved for distinguishing ASD from TD individuals^27–29^ respectively. Unlike resting state imaging data, however, large sample sizes are not often acquired for task related neural imaging studies and multivariate classifications have been less successful. Biological motion has also been shown to classify ASD versus TD individuals with 79% accuracy although no prediction was made for severity of symptomatology for individual patients^30^. Nonetheless, using univariate methods, as discussed above, individuals with ASD show altered activity patterns when viewing simulated faces compared to TD^31,32^. Here we apply a machine learning method, the nested-cross validation approach, to distinguish neural activity related to eye-to-eye contact depending upon whether the individual was TD or an individual with ASD. Using this classification, we further tested the hypothesis that the SVM output using fNIRS measures of neural activity in response to live eye-to-eye contact can be associated with social symptomatology as measured by the ADOS. This approach is suited for relatively sample sizes typical of task-related neuroimaging studies and provides a prediction for each individual rather than a general finding.

## Results

The findings include three sections. First, a description of the neural responses to the tasks that distinguish TD and ASD cohorts. Next, univariate and multivariate analyses are compared. Finally, regression results (ADOS scores) obtained through SVM scores are presented comparing predicted versus measured values.

### General linear model (GLM) compared to principal component analysis (PCA)

**Figure 1:**
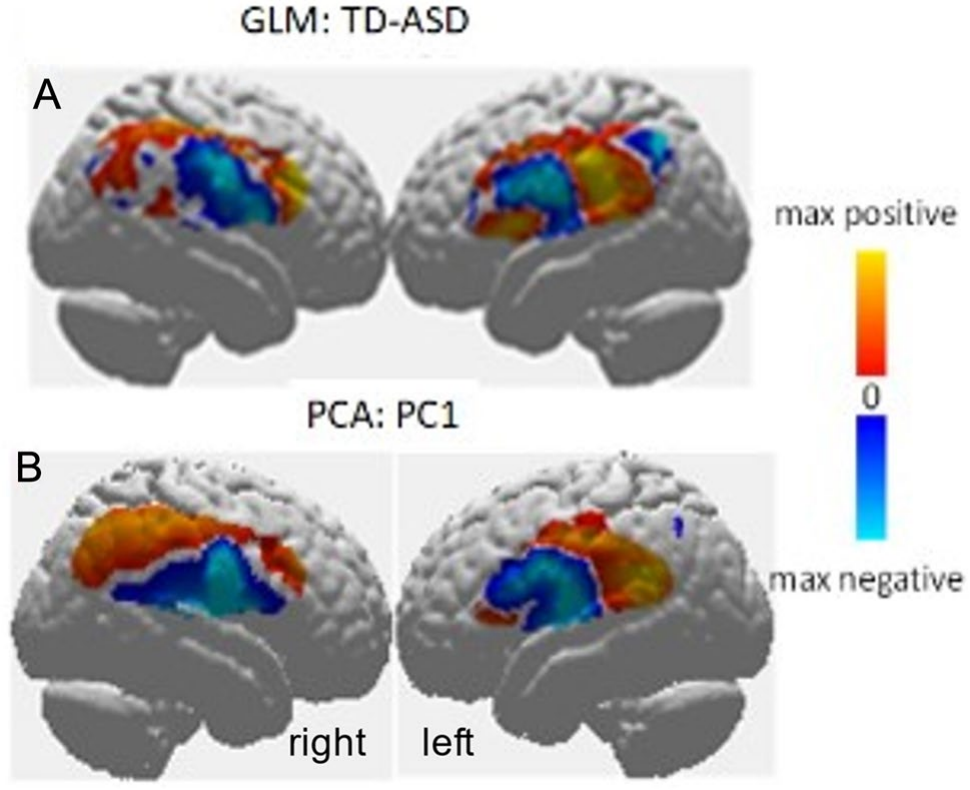
(**A**) The average difference in neural activity determined via GLM for the live face condition between the TD and ASD groups. Red indicates brain regions where the neural activity is greater for the TD group and blue indicates brain regions where the neural activity is greater for the ASD groups. (**B**) The first principal component derived from data for all subjects. Images are “non-thresholded” images based on the beta values of the Hbdiff signal (a signal that incorporates the oxyhemoglobin and the dexyhemoglobin components from the fNIRS acquisition)^33^. We note the similarly between the two localized patterns. Yellow/red indicates brain regions where the first principal component is highest and cyan/blue indicates brain regions where it is lowest. PC1 accounts for 16.3% of the total variance.

Neural responses to the task of eye-to-eye contact are shown in Fig. 1. A rendering of left and right superficial cortical hemispheres compare neural responses from TD and ASD groups during the eye-to-eye task using traditional GLM regression (A) as well as the first principal component of the neural responses (B). These data are derived from the “Hbdiff” signal which incorporates the signal strength of both oxyhemoglobin and deoxyhemoglobin signals^19,33^. Principal components analysis (PCA) was applied to decompose the data into the individual components. While the physiological meaning of any individual principal component is not necessarily related to a specific set of neural responses, the PC1, rendered in Fig. 1B, represents the largest variance in the data. Figure 1A, B both show higher activity in the parietal lobe (dorsal stream). In the case of the contrast (TD > ASD) shown in (Fig. 1A) the yellow/red color indicates regions where the TD signals are greater than the ASD signals, and the cyan/blue color indicates regions where the ASD signals are greater than the TD signals. In Fig. 1B the ASD and TD data are combined, and yellow/red indicates brain regions where the first principal component is highest and cyan/blue indicates brain regions where it is lowest. The similarity between these two methods as illustrated in Fig. 1 suggests that PC1 and GLM comparisons represent similar features between the two groups, and both analysis approaches confirm major differences between the two groups. Subsequent principal components represent additional variance in the data and are more difficult to interpret. For completeness, we have included renders of all individual subject GLM modeled responses in Figure S1 as well as all individual PC patterns in Figure S2.

### Univariate classification of TD versus ASD

Univariate analysis has the advantage of straightforward interpretations based on specific regions and/or neural circuits of interest compared to a multivariate analysis. We first apply a univariate analysis using principal components to separate ASD from TD. The PC1 scores were used to determine a binary decision boundary to classify TD versus ASD. The results of this classification task show an accuracy of 58.3%. However, it is possible that other PCs may better represent the difference in neural responses between the two groups. Other principal components contain additional variance that may improve classification results. In neural imaging it is standard practice to use the best or strongest feature for classification tasks. To determine the best principal component as the input feature we performed a *t* test on all PC scores between the two groups. The PC that had the largest t-score was PC10, rather than PC1. We performed the same classification task using PC10 as the input feature and the classification yielded 55.7% accuracy. These modest accuracies for classification motivate the following multivariate analysis.

Overall, the accuracy in classification between PC10 and PC1 is not improved. This may indicate an inflated t score of PC10 due to the multiple comparison error. These classification accuracies using univariant analyses are compared to multivariate analysis methods. We expect that classification of a complex condition that involves multiple neural mechanisms and data streams, such as live face viewing, will yield a higher classification accuracy.

### Multivariate classification of TD versus ASD

**Figure 2:**
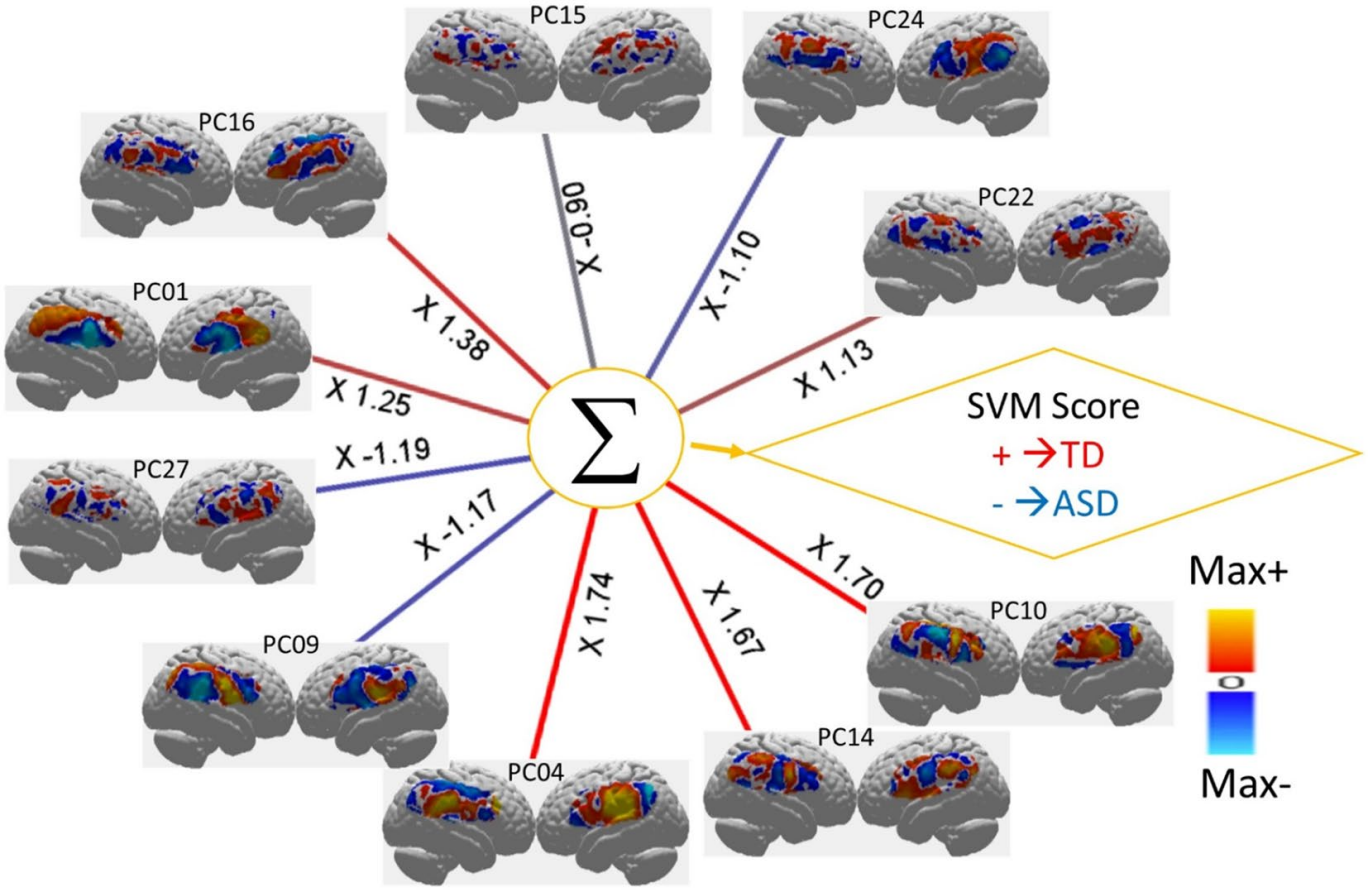
The neural activity pattern for each component in the classifier determined by the Support Vector Machine, SVM. Each pair of brain images is the neural activity of a PC indicated on the insets. The numbers on the edges are the coefficients in Eq. 2. Visualization of the neural activity underlying the classification (the rendered images in the figure) is a unique advantage of the SVM technique. SVM first identifies support vectors, i.e., the closest samples in both groups and then derives a linear classifier based on the support vectors.

The result of a multivariate classifier trained with SVM is shown in Fig. 2. Accuracy was found to be 80.5% with a *p* value < 0.004 estimated using a permutation method (see “Method” section Figs. 5, 6). Input feature selection (principal components served as features) for the SVM classifier was done based on the t-score for the TD versus ASD contrast like the univariate analysis above. Classification using the 10 best PCs (ranked by *t* test) was found to show the highest performance (see “Methods” section, Fig. 6).

### ADOS score prediction

**Figure 3:**
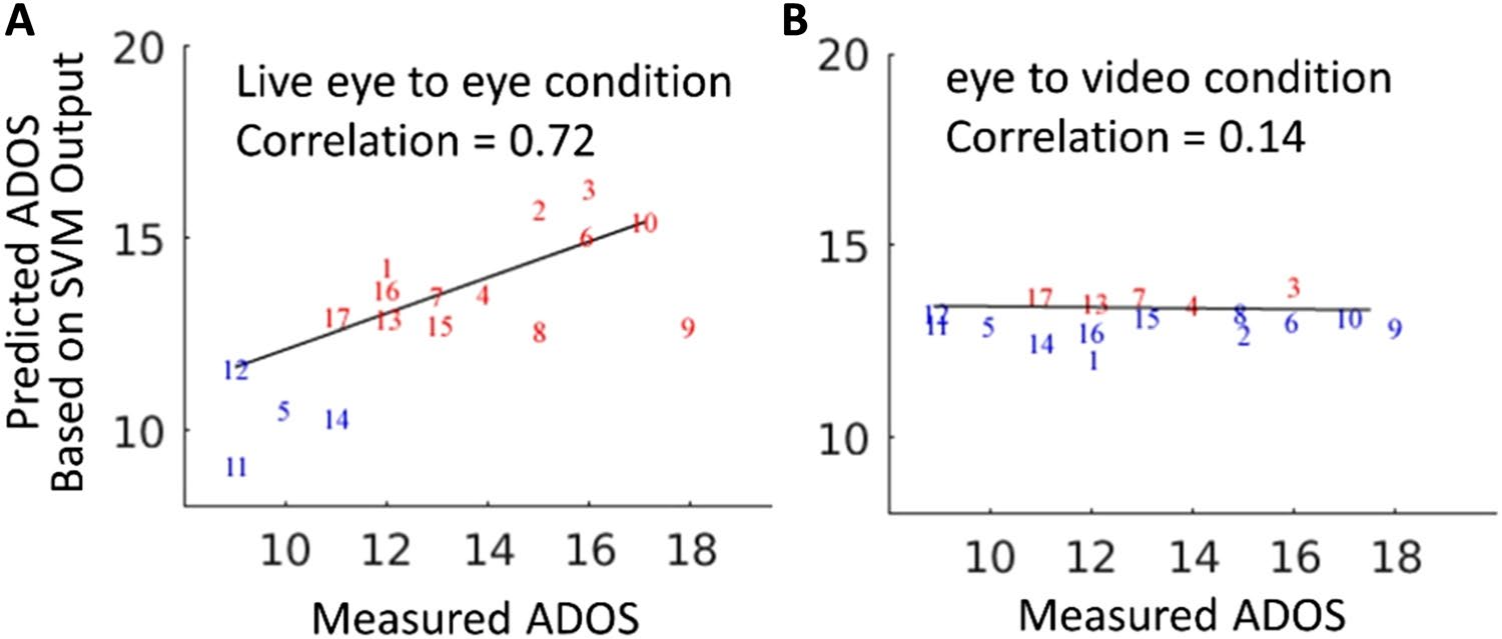
The relationship between the behavioral measure of social symptom severity, ADOS score (X axis), and the predicted ADOS score based on the SVM classification (Y axis), is determined by the brain activity during the live eye to eye condition (**A**) and during the video eye to eye condition (**B**). Increasing ADOS scores indicate increasing symptomatology. Numbers on the scatter plot indicate the individual participants with ASD (see Supplementary Table 1). In Fig. 3 all of the participants are clinically diagnosed with ASD, the red and blue numbers indicate correct and incorrect SVM classifications, respectively. The correlation between observed and predicted, r, is 0.72 for the eye-to-eye condition (**A**) and 0.14 for the video watching condition (**B**). In the eye-to-eye condition (**A**) 4 out of 17 participants with ASD were incorrectly classified as TD, and 12 of the 17 participants were misclassified in the video condition (**B**). To simplify comparison between SVM scores and ADOS scores, we used a linear transform on SVM scores to provide a comparable value for visualization.

To test the hypothesis that SVM identifies the neural activity patterns related to the mechanisms underlying the behavioral symptoms of ASD, we determined the correlation coefficient between the SVM scores based on the classification process and the measured ADOS scores for each of the patients. The ADOS scores were obtained via a standardized, semi-structured interview administered by a clinical psychologist and reflect performance across behavioral domains relevant to autism including as conversation, use of nonverbal communication, and socioemotional insight. ADOS scores were not used during training, and therefore serve as an independent indicator of the success of the SVM classification. In addition, we also performed the same classification and correlation between SVM and ADOS scores on a similar task that included viewing a pre-recorded, non-live face stimulus (eye-to-video condition, Fig. 3A, B, respectively).

Figure 3A shows the individual observed ADOS scores (x-axis) and the predicted ADOS scores (y-axis) based on the SVM classification output with the correlation of r = 0.72 and *p* value < 0.002 indicating that these fNIRS signals are highly correlated to the live face interaction in ASD participants. Figure 3B shows the same comparisons for the same participants based on gaze at a pre-recorded face-video condition with a correlation of r = 0.14 between the observed and predicted ADOS scores. The absence of a significant relationship between predicted and observed SVM output for the face-video condition (Fig. 3B) underscores the specificity for live interactions and direct eye-to-eye contact as shown in Fig. 3A. Although the ADOS scores were not used during SVM training, the SVM output correlates highly with the measured ADOS scores for the live eye-to-eye contact condition indicating generalizability of the SVM model. Comparison of the correlation for the live eye-to-eye condition (3A) and the video eye-to-eye condition (3B) provide further evidence for the real-face effect on neural systems associated with behavioral difficulties in ASD.

## Discussion and conclusion

In this study we hypothesized that the neural activity related to a task involving real eye contact with a human partner would convey sufficient information to classify ASD and TD groups based on functional near-infrared spectroscopic signals. Multivariate classification was found to distinguish TD and ASD groups. The high correlation (r = 0.72) between SVM scores and clinical ADOS measures of social symptomatology suggest an advance for early detection and possible strategies for intervention using these methods. We previously showed that traditional GLM methods of fNIRS data were able to determine differences in neural activity between TD and ASD participants^19^. Here we show the first principal component of fNIRS data has a similar spatial pattern to the GLM result confirming major differences in neural activity between ASD and TD.

Despite rigorous scientific inquiry, objective biomarkers for ASD remain a significant gap in knowledge. These results provide initial evidence for a potential index relevant to clinical classification of single patients with ASD using machine learning and hemodynamic responses recorded during a live eye-to-eye contact. Our results show that the principal components of neural activity (Fig. 2) provide a classifier that predicts clinical symptom severity for individuals with ASD (Fig. 3). This methodology provides a link between neural activity recorded during live eye contact with a partner and clinically measured symptomatology. The Support Vector Machine provides not only binary classification but also outputs a continuous numerical value that is used as the basis for the classification. We utilize this continuous value for comparison to the gold standard metric (individual ADOS scores) for clinical diagnosis. Further development of this approach to improve correlation with clinical measures in pediatric and community samples could provide biological information to the current clinical diagnostic evaluation of autism. These findings also provide insight into the neural mechanism of ASD by relating the neural patterns associated with eye-contact to the classification of TD versus ASD. Finally, the results of this experiment may also serve as a method to develop additional diagnostic tools for other social conditions that show multivariate patterns of brain activity associated with live person-to-person interaction.

The relatively small number of participants in this study as well as the low signal to noise ratio common in functional neural imaging data from task-studies suggest univariate analysis, either using PC1 or the most significant PC (comparing statistical differences between the two groups) can be an appropriate candidate for discriminating the two groups. However, results of univariate analysis yield less than 60% classification accuracy. One contributing factor to this low accuracy is cross-subject variability shown in individual data (Figure S1) and, more importantly, the presumed heterogeneous and multi-factor nature of the neural mechanisms underlying eye-to-eye contact in ASD.

It has been previously suggested that the number of parameters in a learning model should be no more than half the number of samples to achieve generalizable results^34,35^. In this study, we employed two standard approaches to reduce the dimensions of the input for classification. First, we reduced the number of input dimensions of the fNIRS data using PCA into 32 principal components. Secondly, we selected a subset of the strongest of the 32 PCs as input features based on the t-test between ASD and TD for SVM classifier. Since this feature selection process removes independence between testing data and training data, we utilized nested cross validation instead of K-fold cross-validation. Nested cross-validation optimized the SVM model to correct for multiple comparisons problem that may arise due to the small number of subjects and low signal to noise ratio in the fNIRS signals. The SVM model was able to classify the two groups (ASD vs TD) and also able to predict the ADOS scores (r = 0.72) from the classification results in a value that the machine has not been trained to learn in accordance with the standard for generalizabilty^36^.

### Limitations

A challenge in multivariate classification with neural imaging data is the large ratio between the input dimension and number of subjects. Numerous default machine learning tools have been designed for large sample sizes. Acquisition of data based on live eye contact with an in-person human partner is an emerging technology and paradigm and large data sets are not available (another challenge to this methodology). The neural mechanisms of ASD are complex and vary greatly among individuals. The sample size of participants with ASD in this investigation was limited to 17 and the sample size for the TD participants was 19. Not only is statistical power challenged with these sample sizes, but a number of characteristics specific to autism may also be missed. A larger pool of subjects is expected to enhance the robustness of machine learning with more advanced architecture and enhanced representation of the neural mechanisms and variability associated with ASD.

## Methods

### Part 1: Participants, tasks, data acquisition and processing

#### Participants

Data in this study have been published previously and methods are described therein^19^. Participants included 17 Autism Spectrum Disorder (ASD) adults (Table S1, 3 female; mean age 25 ± 4.9 years; 12 right-handed, 3 left-handed, and 2 ambidextrous^37^) whose diagnoses were verified by research-reliable clinician assessments, including the Autism Diagnostic Observation Schedule, 2nd Edition (ADOS-2^38^). Nineteen, typically developed (TD) adults (Table S2, mean age 26 ± 5.8 years; 18 right-handed and 1 ambidextrous) also participated. Groups were matched by age, gender, and IQ. All participants provided written and verbal informed consent in accordance with guidelines and regulations approved by the Yale University Human Investigation Committee (HIC #1512016895) and were reimbursed for their participation.

#### Experimental design

**Figure 4:**
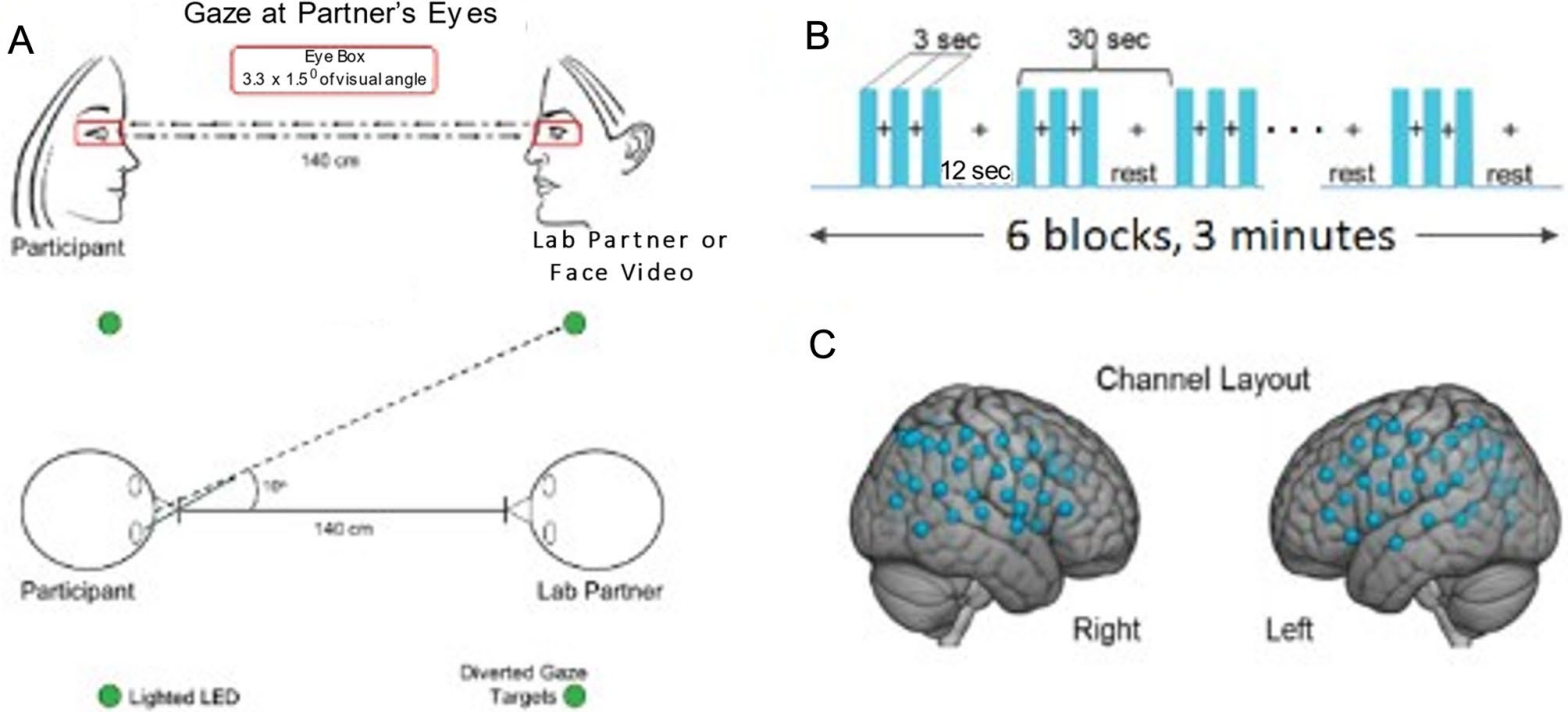
Experimental Setup and Paradigm. (**A**) The side and top views of two partners during the live eye-to-eye (top) and diverted gaze task. One partner was a gender-matched typical confederate. The other partner was either an individual with ASD or was typically developed. (**B**) The time course, of the eye contact task. Blue bars indicate epochs of eye-to-eye contact. (**C**) The locations of the 54 channels applied to both partners.

Dyads consisted of a participant and a confederate. Participants were either individuals with ASD or matched typically developed, TD, individuals. Confederates, referred to as “lab partners”, were gender-matched to the participants and were also typically developed. Dyads were seated 140 cm across a table from each other and were set up with an extended head-coverage fNIRS cap. Each participant was instructed to look either at their partner or a target 10° away from the eye of the partner for 3 s as illustrated in Fig. 4A. There were two conditions, live eye-to-eye and video eye-to-eye where both subjects viewed a video shown with a computer screen instead of a live human partner. Participants viewed partners (human or video) in 3-s epochs for 18 secs and “rested” with diverted eye-gaze for 12 s for a total of 3 min per run, (Fig. 4B)^19,39^ The optodes layout for both hemispheres and both partners is illustrated in Fig. 4C. The Autism Diagnostic Observation Schedule-Second Edition (ADOS-2) was administrated by a trained clinician to assess communication skills, social interaction, and imaginative use of materials for each participant with ASD. The ADOS score is currently the “gold standard” for assessing ASD^40^.

#### Functional NIRS signal acquisition and channel localization

Hemodynamic signals were acquired using a continuous-wave fNIRS system (LABNIRS, Shimadzu Corp., Kyoto, Japan). Fifty four channels were acquired for each TD and ASD participant (Fig. 1C) with a sample rate of 30 sample/s. Montreal Neurological Institute (MNI) coordinates^41^ for each channel and each subject, measured with a three-dimensional (3-D) digitizer (Polhemus Tech, Vermont) and calculated using NIRS-SPM software^42^.

#### fNIRS signal processing

Baseline drift was removed using wavelet detrending provided in NIRS-SPM^42^. In accordance with recommendations for best practices using fNIRS data^43^, global components attributable to blood pressure and other systemic effects^44^ were removed using spatial global component filter^45,46^. In this study, we integrated the oxyhemoglobin and deoxyhemoglobin signals. The local concentrations of task-based oxyhemoglobin and deoxyhemoglobin signals are anticorrelated, and the combination of the two signals is referred to the “Hbdiff” signal^33^. The Hbdiff signal is a hemodynamic response function similar to the Blood Oxygen Level Dependent, BOLD, signal acquired in task-based fMRI, and is considered a preferred approach to processing signals acquired by fNIRS. This is because it includes all of the signals acquired, and also takes into account the relationship between the two signals and the physiological processes from which they originate. Interpretation of this signal is consistent with the interpretation of conventional task-based fMRI signals. However, it is acquired by optical methods rather by magnetic susceptibility. For the general linear model, GLM, analysis, the time series of the eye contact task (Fig. 4B) were convolved with the hemodynamic response function provided from SPM^47^ and then fit to the signals with general linear model method (GLM), providing a beta value as the amplitude of neural activity for each channel. Following the GLM analysis, the beta values were projected onto the MNI brain surface using the “Easytopo” software^48^ based on the locations of the channels for each individual participant.

### Support vector machine (SVM)

A Support Vector Machine (SVM) is considered to be one of the most robust prediction methods based on statistical learning frameworks^49^ and it is commonly applied as a supervised machine learning algorithm used for classification or regression tasks. The main goal for the SVM algorithm is to find the hyperplane that optimally separates different classes in the training data. This is done by maximizing the margin between the decision boundary and the closest data points (i.e. most difficult points) from each class^50^. SVM is well-suited for high-dimensional and complex data such as individual neural imaging data, medical information, and other classes of behavioral and subjective information. Here we apply linear classifications using SVM, as opposed to non-linear methods, as it is the approach of least assumptions.

Previously, we have investigated neural activity in adults with ASD and also adults who were typically developed (TD) during live eye-to-eye contact and have shown using functional near infrared spectroscopy, fNIRS, hypoactivity in the right dorsal parietal visual stream for individuals with ASD^19^. Here, we use the neural and behavioral data from this previous study to test the additional hypothesis that multivariate classification based on the neural activity patterns would (1) distinguish TD versus ASD participants, and also (2) quantitatively reflect individual factors associated with the severity of symptoms assessed on the Autism Diagnosis Observation Schedule, ADOS^40^. In the current study we do not consider ADOS scores during training. An observed association of individual ADOS and SVM scores would be taken as an independent indicator of the extent to which the neural systems that process live eye-to-eye contact are linked to symptomatology.

To achieve high performance using multivariate classification with functional neural imaging data, two challenges must be addressed: overfitting and multiple comparisons with small data sets such as those typically collected using functional neural imaging on clinical populations. Overfitting refers to the fact that, when the number of parameters of any model is greater or close to the number of subjects, a model can be derived to fit any random data set. K-fold cross-validation is a standard practice to control overfitting in multivariate classifications. With respect to neural imaging data, the input dimension or the number of features is far greater than the number of subjects and has low signal to noise ratio (SNR). Therefore, reduction in dimensionality through feature selection is critical for data with small sample sizes.

The problem of multiple comparisons refers to incorrect statistical inference due to choosing a subset of features based on the target of the analysis such as the diagnostic status of ASD or TD. For example, a region showing the greatest average difference between ASD and TD may be at risk for a multiple comparisons error because it is chosen using the diagnostic status (label) of each individual subject. Due to this potential multiple comparisons problem, K-fold cross-validation has been shown to be unreliable for feature selection yielding 75%, instead of chance (50%) level of accuracy on random data with a small number of samples^51–53^. More importantly, the accuracy inflation is not uniform, i.e., certain models can cause more inflation and lead to suboptimal performance. Here, we adopt an established method of nested-cross validation in which the testing data are not used during feature selection and the accuracy on random data is at chance regardless of the number of subjects^54,55^.

### Part 2: Dimensionality reduction and classification methods

#### Principal component analysis (PCA) of spatial features

PCA is a standard technique to reduce the dimension of input data^34^. In this study, the input for PCA is the neural activity of subjects from both groups, rendered on the brain surface (Fig. S1). PCA transforms the raw data matrix with a size of [total number of subjects × number of points in brain surface] into [total number of subject X 32 PC_score] array and each PC is associated with a brain activity pattern shown in Fig. S2 (Eq. 1).

1 $$\left[ {\begin{array}{*{20}l} {image_{subj01} } \hfill \\ {image_{subj02} } \hfill \\ \ldots \hfill \\ {image_{subj36} } \hfill \\ \end{array} } \right]\mathop { < = = > }\limits^{PCA} \left[ {\begin{array}{*{20}l} {PC\,score_{subj01}^{01} } \hfill & \ldots \hfill & {PC\,score_{subj01}^{32} } \hfill \\ {PC\,score_{subj02}^{01} } \hfill & \ldots \hfill & {PC\,score_{subj02}^{32} } \hfill \\ \ldots \hfill & \ldots \hfill & \ldots \hfill \\ {PC\,score_{subj36}^{01} } \hfill & \ldots \hfill & {PC\,score_{subj36}^{32} } \hfill \\ \end{array} } \right]*\left[ {\begin{array}{*{20}l} {PC_{01} } \hfill \\ {PC_{02} } \hfill \\ \ldots \hfill \\ {PC_{32} } \hfill \\ \end{array} } \right]$$

Given the PCs established in Eq. 1, the image for the ith subject can be reconstructed with Eq. 2:

2 $$image_{i} = \sum\limits_{j = 1}^{32} {PC\,score_{i}^{j} *PC_{j} }$$

#### Sorting the principal components, PCs, for feature selection

To determine which PC was most informative a non-paired *t* test between the TD and the ASD groups was performed. The absolute values of the t-scores were used for sorting the PCs from the greatest to the smallest difference between the TD and ASD groups. The results of this comparison are shown in Supplementary Fig. 2.

#### Controlling for multiple comparisons using K-fold cross-validation versus nested cross-validation

**Figure 5:**
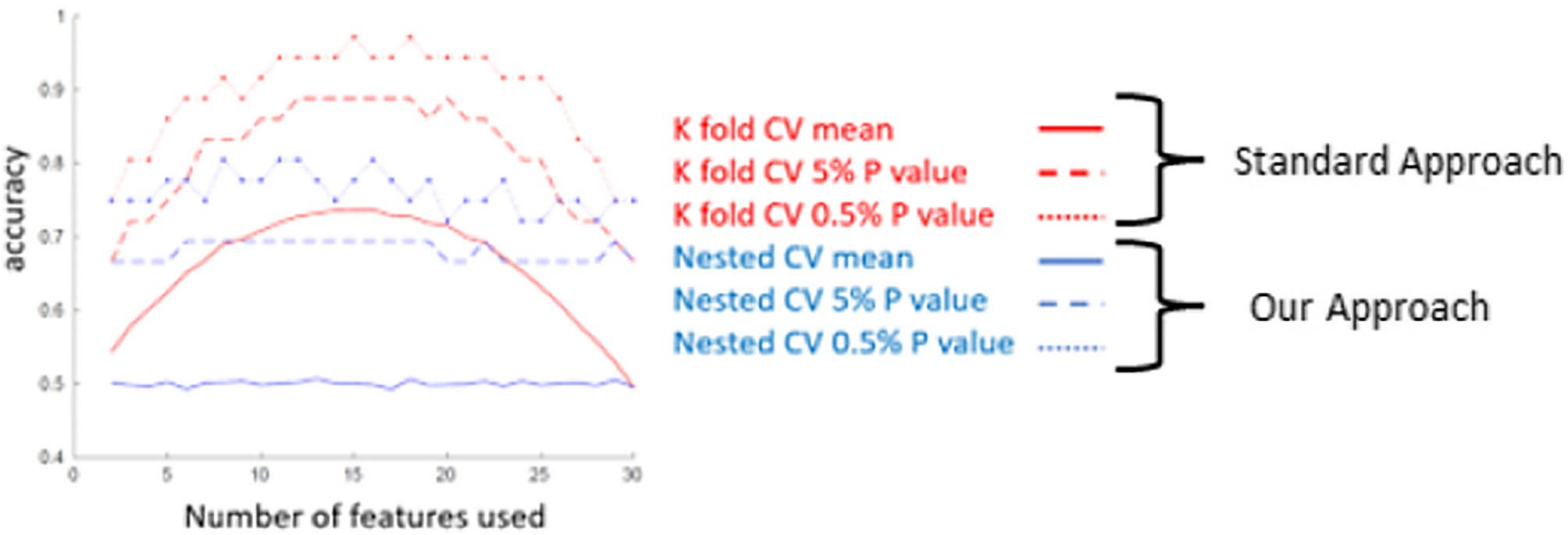
Parameter tuning curve using random permutations. The relationship between the number of used features (PCs) in SPM analyses (x-axis) and the performance of SVM classification (Accuracy) (y-axis) is shown for random classification labels indicating diagnostic status. The red lines are obtained with K-fold cross-validation and the blue lines are obtained with the nested cross-validation. 0.05 and 0.005 confidence intervals were plotted as the dash and dotted lines, respectively, based on 1000 random tests.

For the K-fold cross-validation, the sorting of PCs was done for all the subjects. In contrast, for the nested cross-validation, the sorting of PCs were done using training data, where the testing data was excluded from the *t* test calculation. In both cases, a leave-one-out cross validation was done to measure the accuracy of a classification algorithm. In this study we utilized nested cross-validation instead of K-fold validation because a comparison between the two methods produced results closer to chance (50%) when training data with scrambled labels which is the expected result. See the blue lines in Fig. 5.

**Figure 6:**
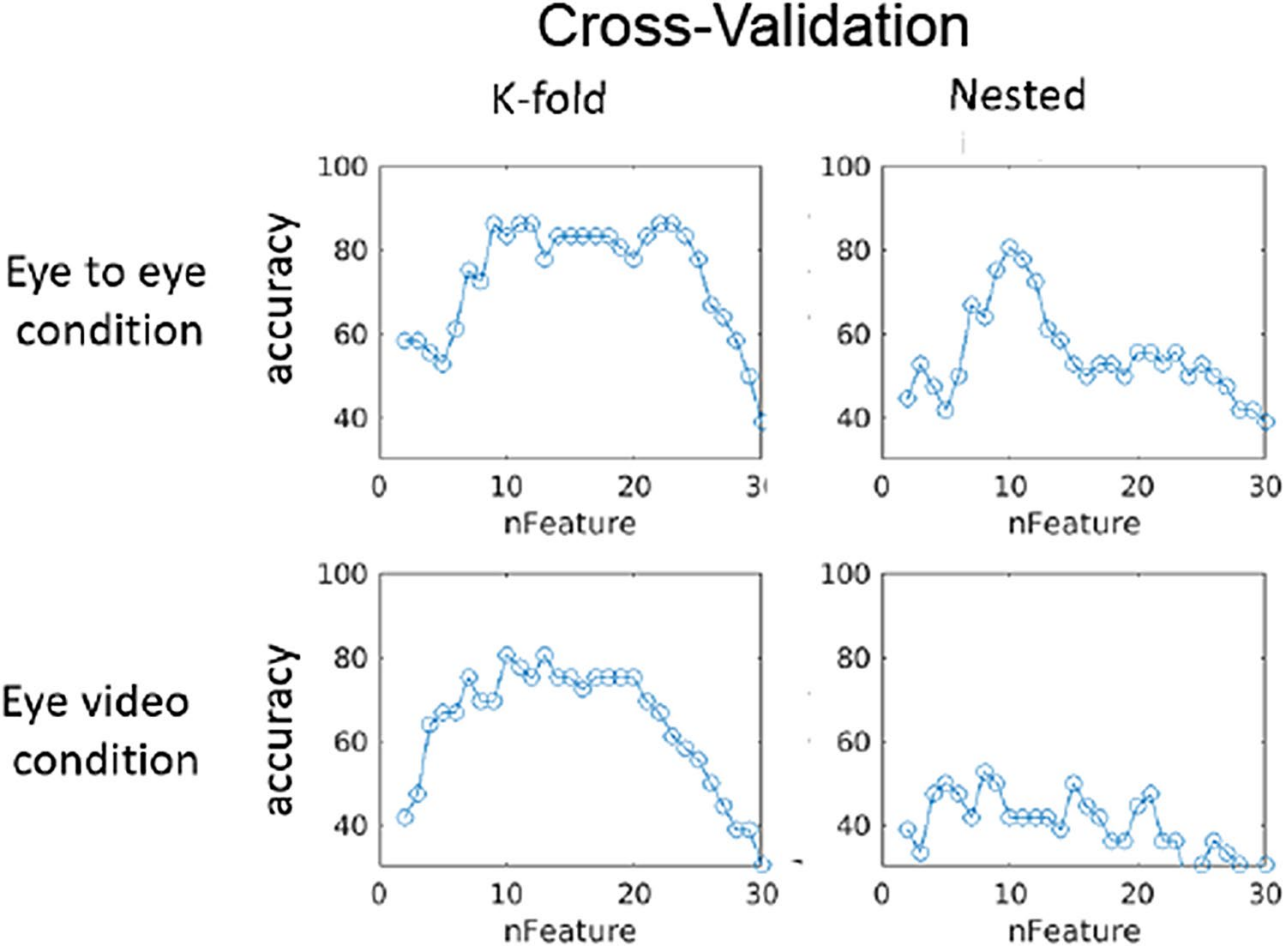
The relationship between the number of used features (PCs) in SPM analyses and the performance of SVM classification (Accuracy) using real data. The left panels are obtained with K-fold cross-validation and the right panels are obtained with the nested cross-validation. The top row is for the eye-to-eye condition and bottom row is for the eye-video condition.

The optimization of the Support Vector Machine (SVM) was performed by varying the number of best Principal Components (PCs) used as input to the SVM. The result for the Eye-to Eye and the Video-Eye gaze conditions are shown for comparison in Fig. 6. The right top panel of shows the result of the parameter tuning curve and indicates that the ten best PCs as the input to the SVM (x-axis) have the best performance using the nested cross-validation method. As can be seen from the figure, the optimal number of parameters is 10, which is about one-third of the number of subjects and is considered appropriate for a statistical model. Note that the tuning curves of K-fold cross validation (left panels) show neither specificity for condition nor specificity for the number of features as the optimal parameter for SVM. Therefore, we conclude that the nested cross-validation is a preferred method for SVM.

#### Univariate classification

We compared the classification of previously collected fNIRS data using both univariate and multivariate techniques to determine if a small neuroimaging data set would gain additional clinical benefit by using multivariate analyses. Conventional classification based on univariate analysis uses one dimensional data. Linear classifiers are expressed as

3 $$Y = wX + b\left| {\begin{array}{*{20}l} {Y > 0 - \to TD} \\ { Y < 0 - \to ASD} \\ \end{array} } \right.$$

where *Y* is the diagnostic label, *X* is the measurement with one value per subject, *w* is either 1 or − 1 and *b* is the threshold. Here the training of the model is finding the b value that results in highest accuracy for the training data set and then apply the formula to the testing data. An example of X could be either brain activity amplitude of the channel or the principal component of brain activities that shows the largest difference between TD and ASD. In this study, we used either PC1 or the PC with the largest difference between TD and ASD groups, noted as the “best PC” to determine univariate classification accuracy. We employed a logistic regression method to compare classification accuracy between the groups using nested cross-validation to compensate for multiple comparisons. The nested cross-validation suggests that the “best” PC, the PC with the largest t-test between the two groups, is not the most informative one due, in part, to the multiple comparison error.

#### Multivariate classification

The multivariate classification tool used in this study is the support vector machine (SVM, fitcsvm function with linear kernel^56^) provided in the Statistics and Machine Learning Toolbox in MATLAB 2010. Any input for SVM with dimensions that are close to or greater than the number of subjects may result in bias and overfitting^34,35^. Therefore, one fundamental step in multivariate classification is feature reduction and selection. The input features in multivariate classification were the principal component (PC scores), sorted by the statistical difference between the two groups. Optimization parameters are the number of best PCs being used. To provide a statistical measure of performance, we look at the p-value of the SVM result. SVM was trained 1000 times with a data set containing random diagnostic labels. The P value is calculated based as the percentage of the random data sets that have higher accuracy then accuracy of the real data set. As noted above, with K-fold cross-validation, an 88% accuracy is needed for reaching statistical significance. In contrast, with nested cross-validation, the statistical criterion is more aligned with typical expectations for significance (Fig. 5).

#### Prediction of ADOS scores

The output of a SVM binary classifier (SVM score) is a continuous value, and was used to determine correlation with the participant ADOS scores. Although multivariant regression tools could be trained with ADOS score makes prediction of ADOS, in this study we did not use the ADOS scores for training. Since the ADOS scores have not been trained for SVM, the prediction of ADOS is also a test of generalizability of out model.

### Ethical approval and guidelines

All participants provided written and verbal informed consent in accordance with guidelines and regulations approved by the Yale University Human Investigation Committee (HIC #1512016895) and were paid for their participation. Assessment of the capacity of participants with ASD to give informed consent was provided by a consensus of trained professional staff who monitored the process and confirmed verbal and non-verbal responses. In order to assure that participants were comfortable during the experimental procedure, participants with ASD were accompanied at all times by a member of the clinical team, who continuously evaluated their sustained consent to participate.

### Supplementary Information

Supplementary Information.

## Data Availability

Data used in the study are available on Dryad at DOI: https://doi.org/10.5061/dryad.w6m905qvp. The code used to process the data is available at https://github.com/xz63/SVM-indepedent-cross-validation.
