# Supplementary material for: Support vector machine prediction of individual Autism Diagnostic Observation Schedule (ADOS) scores based on neural responses during live eye-to-eye contact
Source: Sci Rep. 2024 Feb 8;14:3232. doi: 10.1038/s41598-024-53942-z (PMC10853508; doi:10.1038/s41598-024-53942-z)
Supplement: Supplementary file 1 — Supplementary Information. [file 41598_2024_53942_MOESM1_ESM.docx]

**Supplementary Material**

**Title: Support Vector Machine Prediction of Individual Autism Diagnostic Observation Schedule (ADOS) Scores Based on Neural Responses During Live Eye-to-Eye Contact**

**Authors:**

Xian Zhang, J. Adam Noah, Rahul Singh, James C. McPartland and *Joy Hirsch


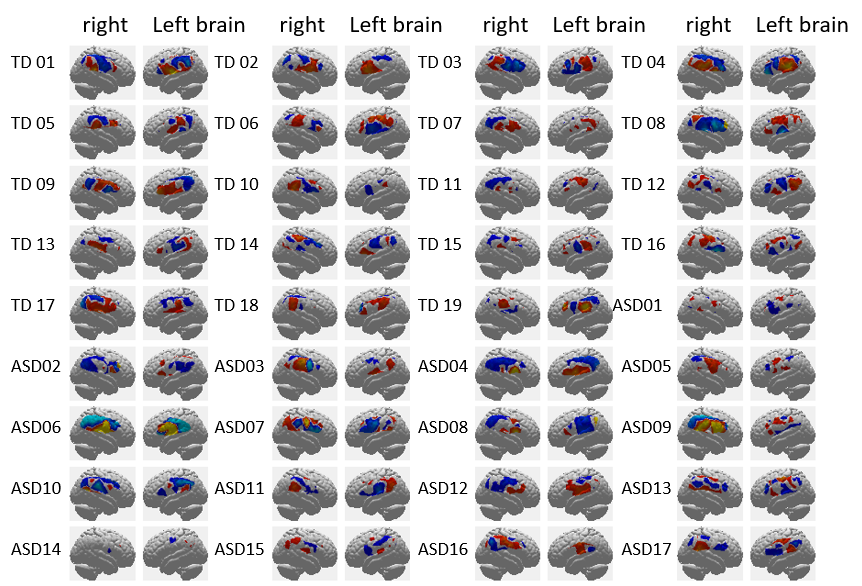


**Figure S1.** The neural activity, recorded as the raw HBdiff signal (a signal that combines both the oxyhemoglobin and the deoxyhemoglobin signal components (Tachtsidis, et al, 2009), for each individual subject (n=36) during the eye-to-eye task. Participants are identified as TD, typically developed, and as ASD, participants with ASD. The color calibration is same for all subjects, a less saturated color indicates lower magnitude of neural activity. Signals are relative to baseline: positive correlation with task (red) and negative (blue) may correspond to default activity. See Fig 1A for the group contrast results.

Unthresholded neural activity for each participant during the live eye-to-eye task for Typically Developed (TD) and participants with Autism Spectrum Disorder (ASD).

**Principal Components (PCs) derived from the combined brain activity of all participants shown in Figure S1 above.**

.


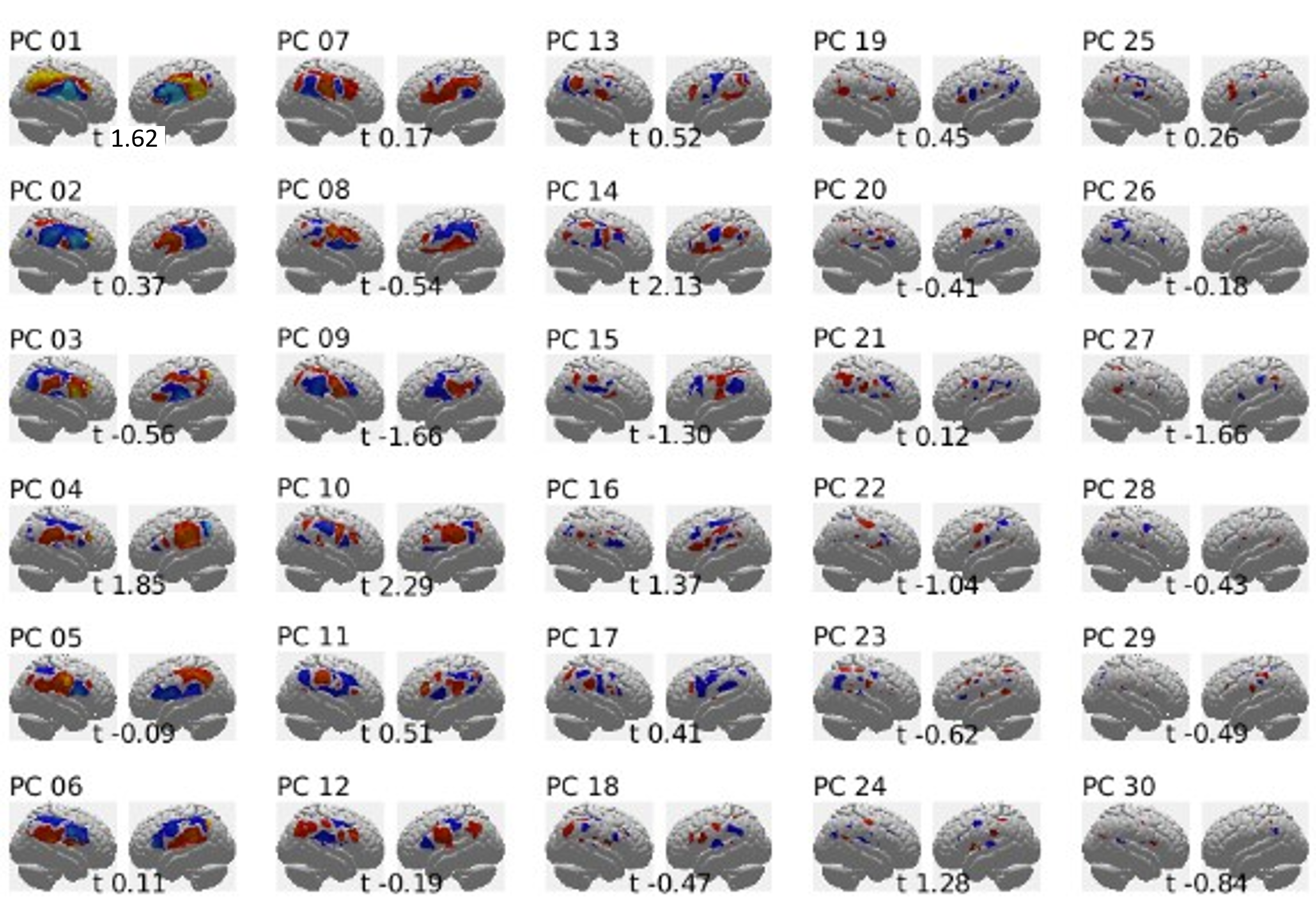


**Figure S2.** The neural activity patterns for PCs for the live eye-to-eye condition are shown. A less saturated color indicates lower magnitude of neural activity. The PC index is shown in the title of each plot, which is inversely related to the rank of a PC in the signal strength. For each image, the t value indicates the t statistic for TD greater than ASD comparison. A negative t value indicates that ASD has greater activity for that PC than TD. See Figure 1B for the group results.

| **Table S1.** Study Participants and Behavioral Test Scores: ASD | | | | | | | | | | | | |
| --- | --- | --- | --- | --- | --- | --- | --- | --- | --- | --- | --- | --- |
| **ID** | **M/F** | **Age** | | **FSIQ-4** | **AQ** | **BAPQ** | **SRS-2** | **BAI** | **STAI** | **LSAS** | **ADOS-2** | **Eye tracking** |
| **1** | Male | | 28-32 | 114 | 26 | 123 | 54 | 16 | 21 | 30 | 12 | Yes |
| **2** | Male | | 33-37 | 104 | 23 | 121 | 30 | 8 | 31 | 0 | 15 | * |
| **3** | Male | | 28-32 | 112 | 30 | 137 | 82 | 11 | 27 | 46 | 16 | * |
| **4** | Male | | 23-27 | 108 | 15 | 102 | 53 | 21 | 30 | 56 | 14 | Yes |
| **5** | Male | | 23-27 | 97 | 15 | 99 | 41 | 19 | 29 | 43 | 10 | Yes |
| **6** | Male | | 23-27 | 90 | 26 | 131 | 132 | 41 | 53 | 79 | 16 | * |
| **7** | Male | | 28-32 | 113 | 15 | 94 | 68 | 7 | 44 | 44 | 13 | * |
| **8** | Male | | 18-22 | 95 | 24 | 117 | 79 | 24 | 37 | 38 | 15 | Yes |
| **9** | Male | | 18-22 | 101 | 30 | 140 | 96 | 27 | 46 | 85 | 18 | Yes |
| **10** | Female | | 28-32 | 110 | 20 | 124 | 70 | 9 | 61 | 34 | 17 | Yes |
| **11** | Female | | 18-22 | 128 | 32 | 121 | 68 | 4 | 30 | 32 | 9 | Yes |
| **12** | Male | | 18-22 | 124 | 25 | 128 | 60 | 1 | 35 | 44 | 9 | Yes |
| **13** | Male | | 18-22 | 107 | 14 | 95 | 34 | 9 | 55 | 5 | 12 | Yes |
| **14** | Male | | 18-22 | 121 | 30 | 155 | 103 | 17 | 41 | 77 | 11 | Yes |
| **15** | Male | | 23-27 | 112 | 20 | 117 | 36 | 3 | 23 | 3 | 13 | Yes |
| **16** | Male | | 28-32 | 101 | 47 | 189 | 153 | 25 | 67 | 126 | 12 | * |
| **17** | Female | | 28-32 | 109 | 23 | 94 | 65 | 15 | 33 | 38 | 11 | Yes |
| *Data unavailable | | | | | | | | | | | | |

**Table S1.** Demographic information for participants with Autism Spectrum Disorder (ASD) participants. Assessment measures include the Autism-Spectrum Quotient test (AQ, total scores); Broad Autism Phenotype Questionnaire (BAPQ, total scores); Social Responsiveness Scale, Second Edition (SRS-2, raw scores); Beck Anxiety Inventory (BAI, total scores); State-Trait Anxiety Inventory (STAI; total state anxiety scores); Liebowitz Social Anxiety Scale (LSAS, total scores); and the Autism Diagnostic Observation Schedule (ADOS-2, total scores). The Wechsler Abbreviated Scale of Intelligence, 2^nd^ Edition (WASI-II) was administered to estimate full-scale intelligence quotient scores based on four subtests (FSIQ-4). *Indicates data were not acquired.

| **Table 2.** Study Participants and Behavioral Test Scores: TD | | | | | | | | | | |
| --- | --- | --- | --- | --- | --- | --- | --- | --- | --- | --- |
| **ID** | **M/F** | **Age** | **FSIQ-4** | **AQ** | **BAPQ** | **SRS-2** | **BAI** | **STAI** | **LSAS** | **Eye tracking** |
| **1** | Male | 18-22 | * | 12 | 77 | 52 | 10 | * | 23 | Yes |
| **2** | Male | 18-22 | 123 | 23 | 118 | 55 | 4 | 39 | 52 | Yes |
| **3** | Male | 28-32 | 113 | 6 | 85 | 9 | 4 | 26 | 24 | Yes |
| **4** | Male | 23-27 | * | * | 72 | 16 | * | * | * | Yes |
| **5** | Female | 23-27 | 129 | 6 | 70 | 20 | 1 | 25 | 23 | Yes |
| **6** | Female | 23-27 | 114 | 8 | 79 | 27 | 5 | 28 | 43 | Yes |
| **7** | Female | 28-32 | 103 | 1 | 53 | 6 | 3 | 21 | 22 | Yes |
| **8** | Male | 28-32 | * | 26 | 109 | 48 | 0 | 23 | 23 | Yes |
| **9** | Female | 18-22 | 79 | 32 | 121 | 64 | 5 | 37 | 44 | Yes |
| **10** | Female | 23-27 | 119 | 22 | 111 | 37 | 26 | 62 | 60 | Yes |
| **11** | Female | 28-32 | 107 | 14 | 88 | 36 | 2 | 29 | 63 | * |
| **12** | Male | 28-32 | 111 | 10 | 73 | 25 | 5 | 28 | 27 | * |
| **13** | Female | 23-27 | 126 | 14 | 93 | 43 | 10 | 38 | 12 | * |
| **14** | Male | 18-22 | * | 25 | 105 | 84 | 7 | 27 | 59 | Yes |
| **15** | Female | 23-27 | 122 | 7 | 79 | 12 | 0 | 26 | 32 | Yes |
| **16** | Male | 38-42 | * | 21 | 124 | 63 | 9 | 56 | 56 | Yes |
| **17** | Male | 18-22 | * | 19 | 96 | 45 | 0 | 22 | 45 | * |
| **18** | Male | 28-32 | 119 | 19 | 89 | 38 | 12 | 26 | 46 | Yes |
| **19** | Male | 38-42 | 121 | 10 | 73 | 31 | 0 | 20 | 18 | Yes |
| *Data unavailable | | | | | | | | | | |

**Table S2.** Demographic information for Typically-Developed (TD) participants. Assessment measures include the Autism-Spectrum Quotient test (AQ, total scores); Broad Autism Phenotype Questionnaire (BAPQ, total scores); Social Responsiveness Scale, Second Edition (SRS-2, raw scores); Beck Anxiety Inventory (BAI, total scores); State-Trait Anxiety Inventory (STAI; total state anxiety scores); and the Liebowitz Social Anxiety Scale (LSAS, total scores). The Wechsler Abbreviated Scale of Intelligence, 2^nd^ Edition (WASI-II) was administered to estimate full-scale intelligence quotient scores based on four subtests (FSIQ-4). *Indicates data were not acquired.
